# Plasma Heme Oxygenase-1 Concentration in Relation to Impaired Glucose Regulation in a Non-Diabetic Chinese Population

**DOI:** 10.1371/journal.pone.0032223

**Published:** 2012-03-12

**Authors:** Wei Bao, Shuang Rong, Muxun Zhang, Xuefeng Yu, Yanting Zhao, Xiao Xiao, Wei Yang, Di Wang, Ping Yao, Frank B. Hu, Liegang Liu

**Affiliations:** 1 Department of Nutrition and Food Hygiene, School of Public Health, Tongji Medical College, Huazhong University of Science and Technology, Wuhan, People's Republic of China; 2 China Ministry of Education Key Lab of Environment and Health, School of Public Health, Tongji Medical College, Huazhong University of Science and Technology, Wuhan, People's Republic of China; 3 Department of Internal Medicine, Institute of Endocrinology, Tongji Hospital, Tongji Medical College, Huazhong University of Science and Technology, Wuhan, People's Republic of China; 4 Departments of Nutrition and Epidemiology, Harvard School of Public Health, Boston, Massachusetts, United States of America; Universidad Peruana Cayetano Heredia, Peru

## Abstract

**Background:**

Our previous study has recently shown that plasma heme oxygenase-1 (HO-1), a stress-responsive protein, is elevated in individuals with type 2 diabetes. The current study aimed to examine the association between plasma HO-1 concentration and impaired glucose regulation (IGR) in non-diabetic individuals.

**Methods:**

We conducted a case-control study including a total of 865 subjects (262 IGR individuals and 603 healthy controls) in a Chinese population. Basic characteristics were collected by questionnaire and standardized anthropometric measurements. Plasma HO-1 concentration was determined by ELISA.

**Results:**

Plasma HO-1 concentration was significantly increased in IGR individuals compared with healthy controls (1.34 (0.81–2.29) ng/ml *vs* 0.98 (0.56–1.55) ng/ml, *P*<0.001). After adjustment for age, sex, and BMI, the ORs for IGR in the highest quartile of plasma HO-1 concentrations, compared with the lowest, was 3.42 (95% CI 2.11–5.54; *P* for trend <0.001). The trend remained significant even after additional adjustment for smoking, alcohol drinking, hypertension, family history of diabetes, lipid profiles and C-reactive protein. In the receiver-operating characteristic curve analysis, addition of plasma HO-1 concentration to a model with known risk factors yielded significantly improved discriminative value for IGR (area under the curves 0.75 (95% CI 0.71–0.78) *vs*. 0.72 (95% CI 0.69–0.76); *P* for difference = 0.026).

**Conclusions:**

Elevated plasma HO-1 concentration is significantly associated with increased ORs for IGR. However, its clinical utility should be validated in further studies, especially in prospective cohort studies.

## Introduction

Individuals with impaired glucose regulation (IGR), including impaired fasting glucose (IFG) and impaired glucose tolerance (IGT), has been demonstrated to exert relatively high risk for the future development of type 2 diabetes mellitus (T2DM) [1] as well as cardiovascular disease (CVD) [2]. Although the pathogenesis of IGR remains unclear, enhanced oxidative stress in the form of lipid peroxidation and DNA oxidative damage in IGR is found in previous studies [3], [4]. In fact, oxidative stress has been implicated in insulin resistance and beta-cell dysfunction which are essential clinical characteristics of IGR and T2DM [3]. Heme oxygenase-1 (HMOX1 or HO-1), also termed as heat shock protein 32, is the inducible isoform of heme oxygenase that catalyzes the NADPH-dependent decomposition of heme to carbon monoxide (CO), ferrous iron, and biliverdin [5]. HO-1 expression is highly responsive to a broad spectrum of chemical and physical stress agents, such as hydrogen peroxide, heavy metals, UVA irradiation, hypoxia, hyperoxia, pro-inflammatory cytokines and heme itself [6], [7]. Therefore, HO-1 as a stress-inducible protein has been suggested to be a sensitive and reliable marker of oxidative stress status [6], [8].

Recently, we have reported that plasma level of HO-1 is significantly elevated in T2DM patients compared with non-diabetic controls (including IGR individuals and healthy controls) [9]. In addition, we have found that plasma HO-1 levels are significantly correlated with plasma glucose concentrations (FPG and OGTT2h), HOMA-beta, and HOMA-IR [9], which makes us think whether plasma HO-1 levels are significantly increased in non-diabetic individuals with moderate hyperglycemia but under the cut-point of diabetes diagnosis. Similar elevation of circulating HO-1 levels has also been found in CVD [10], [11] and other chronic diseases [12], [13], [14]. However, the association between plasma HO-1 concentration and IGR has not yet been investigated.

Thus, in this study, we aimed to assess the association between plasma HO-1 concentration and IGR without or with adjustment for known risk factors for IGR. Additionally, we compared the discriminative value of models for IGR without or with plasma HO-1.

## Methods

### Study population

A total of 865 participants, including 262 IGR individuals and 603 healthy controls, were recruited for the current study. Some of these participants are included in our previous study [9] as non-diabetic controls. IGR individuals were consecutively recruited from those attending the outpatient clinics of Department of Endocrinology of Tongji Hospital affiliated to Tongji Medical College during the period of December 2004 to December 2007. Healthy controls were drawn from an unselected group of population that underwent for a routine health examination in the same hospital during the same period. IGR, including impaired fasting glucose (IFG) and impaired glucose tolerance (IGT), was diagnosed in accordance with the criteria recommended by World Health Organization in 2006 incorporating both fasting plasma glucose (FPG) and a 2-h oral glucose tolerance test (OGTT) [15]. IFG was defined as 6.1 mmol/L≤FPG<7.0 mmol/L and OGTT2h<7.8 mmol/L; IGT was defined as FPG<7.0 mmol/L and 7.8 mmol/L≤OGTT2h<11.1 mmol/L; and healthy control was defined as FPG<6.1 mmol/L and OGTT2h<7.8 mmol/L. For both the IGR cases and controls, we restricted the study subjects to only individuals who were aged ≥30 years, BMI<40 kg/m^2^, no early history of diagnosed diabetes, nor any other clinically systemic diseases, acute or chronic inflammatory diseases, acute respiratory infection, known cardiovascular disease or cancer. The study protocol was approved by Medical Ethics Committee of Tongji Medical College according to the declaration of Helsinki and written informed consent was obtained from all individuals.

As previously described [4], [9], information about age, sex, smoking, alcohol drinking, hypertension, and family history of diabetes in their first-degree relatives were collected by questionnaire survey. Anthropometric measurements including height (m), weight (kg) and blood pressure (mmHg) were performed using standardized techniques. Body mass index (BMI) was calculated as weight (kg)/square of height (m^2^).

### Laboratory Measurements

Antecubital venous blood samples were drawn into heparinized tubes from all the participants in the morning after an overnight fast. The participants were asked to sit in the upright position to ensure minimal venous occlusion time. For the OGTT test, 75 g oral glucose dissolved in 250 to 300 ml water were asked to be consumed in no more than 5 minutes. Plasma samples were separated and retained for analysis of biochemical parameters, including fasting plasma glucose (FPG), fasting plasma insulin (FPI), 2-h post-glucose load (OGTT2h), total cholesterol (TC), triglycerides (TG) and high-density lipoprotein cholesterol (HDL-C), as previously described [9]. Intra- and inter-assay coefficients of variation were <4% for all these assays. Homeostasis model assessment of beta cell function (HOMA-beta) and insulin resistance (HOMA-IR) were employed to assess the status of insulin secretion and insulin action, respectively. HOMA-beta = 20×FPI (µU/ml)/[FPG (mmol/L)-3.5], HOMA-IR = FPG (mmol/L)×FPI (µU/ml)/22.5 [16].

Plasma HO-1 and C-reactive protein (CRP) concentration were determined by Human HO-1 ELISA Kit (EKS-800, Stressgen/Assay Designs, Ann Arbor, MI, USA) and Human CRP Quantikine ELISA kit (SCRP00, R&D Systems, Minneapolis, MN, USA), respectively. The intra-assay and inter-assay coefficients of variation of these kits have been determined to be <10%.

### Statistical analysis

Statistical analyses were performed using Stata 11.0 (Stata Corp., College Station, TX, USA). Descriptive statistics were calculated for all demographic and clinical characteristics of the study subjects. Comparisons between IGR cases and controls were performed by Chi-square (categorical variables), t test (continuous variables, normal distribution) or Mann-Whitney U test (continuous variables, skewed distribution).

Multivariate logistic regression analysis was used to evaluate the independent association of plasma HO-1 concentration with the likelihood of IGR. Hosmer-Lemeshow goodness-of-fit tests were used to evaluate the appropriate model fit. For calculation of the odds ratios (ORs) for IGR, plasma HO-1 concentrations were categorized in quartiles according to the control group: category 1, <0.56 ng/ml; category 2, 0.56–0.98 ng/ml, category 3, 0.98–1.55 ng/ml and category 4, ≥1.55 ng/ml. Crude and adjusted ORs for IGR were calculated, respectively. To estimate the discriminative value of plasma HO-1 concentrations on IGR, receiver-operating characteristic (ROC) curves were plotted and corresponding areas under the curve (AUC) were compared using a model with known risk factors (Model A, including age, sex, BMI, alcohol consumption, smoking, hypertention and family history of diabetes) and another model with plasma HO-1 concentrations added in (Model B, including Model A Plus HO-1).

All reported *P* values were 2-sided and *P*<0.05 was considered to be statistically significant.

## Results

### Demographic and clinical characteristics of the study subjects

As shown in Table 1, the individuals with IGR, compared to healthy controls, had higher body mass index (BMI), higher prevalence of hypertension and family history of diabetes, higher levels of FPG, OGTT2h, HOMA-IR, TG and CRP, and lower levels of HOMA-beta and HDL-C.

**Table 1:** Demographical and Clinical Characteristics of the Study Subjects.

Characteristics	IGR Cases	Controls	*P* Value
Number of Subjects	262	603	
Age, years	50.11 (11.8)	49.83 (10.02)	0.737
Male, n (%)	141 (53.82)	361 (59.87)	0.100
BMI, kg/m^2^	25.00 (23.31–26.73)	23.23 (21.30–24.98)	<0.001
Smoking, n (%)	61 (23.28)	207 (34.33)	0.001
Alcohol, n (%)	58 (22.14)	194 (32.17)	0.003
Family History of Diabetes, n (%)	39 (14.89)	35 (5.80)	<0.001
Hypertention, n (%)	85 (32.44)	135 (22.39)	0.002
FPG (mmol/L)	5.97 (0.64)	4.81 (0.66)	<0.001
FPI (µU/ml)	9.60 (6.68–13.16)	6.52 (4.76–8.52)	<0.001
OGTT2h (mmol/L)	9.22 (8.45–10.12)	7.47 (7.09–7.76)	<0.001
HbA1c (%)	5.89 (0.57)	5.50 (0.33)	<0.001
HOMA-beta	80.69 (50.55–119.67)	100.25 (71.71–137.38)	<0.001
HOMA-IR	2.52 (1.70–3.67)	1.40 (0.94–1.87)	<0.001
TC (mmol/L)	4.40 (3.67–5.16)	4.36 (3.87–4.95)	0.522
TG (mmol/L)	1.41 (1.00–1.97)	1.08 (0.67–1.53)	<0.001
HDL-C (mmol/L)	1.22 (1.07–1.44)	1.28 (1.13–1.46)	0.040
CRP (ng/ml)	12.01 (4.47–22.75)	8.55 (3.97–14.61)	<0.001

Abbreviations: IGR, impaired glucose regulation; BMI, body mass index; FPG, fasting plasma glucose; FPI, fasting plasma insulin; OGTT2h, 2-h post-glucose load; HOMA-beta, homeostasis model assessment of beta cell function; HOMA-IR, homeostasis model assessment of insulin resistance; TC, total cholesterol; TG, triglycerides; HDL-C, high-density lipoprotein cholesterol; CRP, C-reactive protein.

Data are presented as number (percentage) for categorical data, mean (standard deviation) for parametrically distributed data or median (interquartile range) for nonparametrically distributed data.

### Association between plasma HO-1 levels and odds of IGR

**Figure 1:**
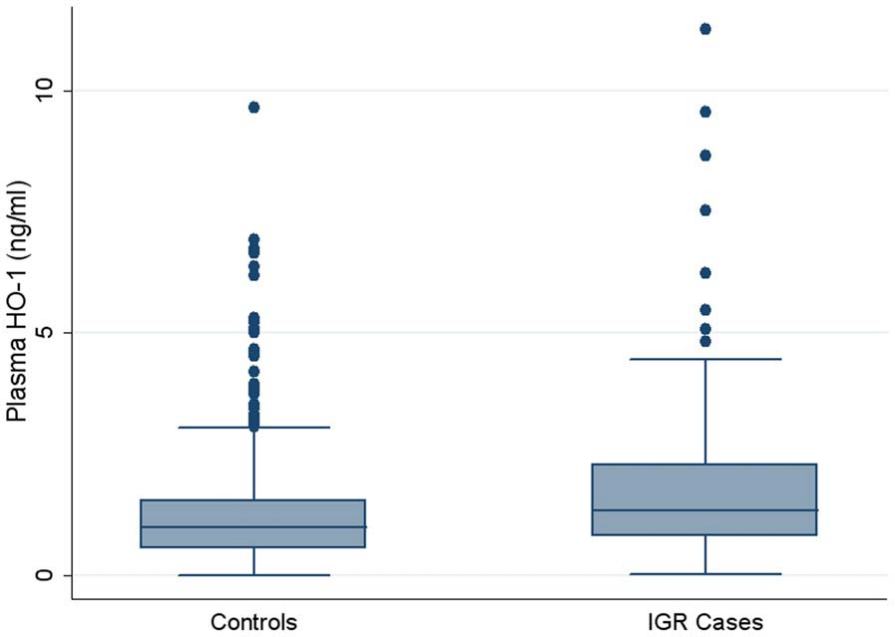
Plasma HO-1 concentrations in IGR individuals and controls. Plasma HO-1 concentration was significantly increased in individuals with IGR compared with controls (1.34 (0.81–2.29) ng/ml vs 0.98 (0.56–1.55) ng/ml, respectively; P<0.001).

Plasma HO-1 concentration was significantly increased in individuals with IGR compared with controls (Median (interquartile range, IQR) 1.34 (0.81–2.29) ng/ml vs 0.98 (0.56–1.55) ng/ml, respectively; P<0.001) (Figure 1).

In the logistic regression analysis, we observed increased ORs for IGR associated with higher level of the plasma HO-1 (Table 2). Individuals in the highest quartile of plasma HO-1 levels had a significantly increased ORs compared with those in the lowest quartile (crude OR 3.45, 95% confidence interval (CI) 2.19–5.42, *P* for trend <0.001). Adjustment for age, sex and BMI (adjusted OR 3.42, 95% CI 2.11–5.54; *P* for trend <0.001; Model 1) or further adjustment for smoking, alcohol drinking, hypertension and family history of diabetes (adjusted OR 3.39, 95% CI 2.08–5.54; *P* for trend <0.001; Model 2) did not alter the results. The trend remained significant even after additional adjustment for lipid profiles (adjusted OR 3.29, 95% CI 2.00–5.43; *P* for trend <0.001; Model 3) and further C-reactive protein (adjusted OR 3.12, 95% CI 1.89–5.16; *P* for trend <0.001; Model 4).

**Table 2:** Odds Ratios (95% CI) of IGR Prevalence, by Quartile of Plasma HO-1 Levels.

Variable	Quartile of Plasma HO-1 Levels				*P* Value for Trend
	1 (Lowest)	2	3	4 (Highest)	
Plasma HO-1 Levels, ng/ml	<0.56	0.56–0.98	0.98–1.55	≥1.55	/
Cases/Controls, n/n	32/150	54/152	65/150	111/151	/
Crude OR (95% CI)	1	1.67 (1.02–2.72)	2.03 (1.26–3.28)	3.45 (2.19–5.42)	<0.001
Adjusted OR (95% CI), Model 1	1	1.95 (1.16–3.29)	2.37 (1.42–3.93)	3.42 (2.11–5.54)	<0.001
Adjusted OR (95% CI), Model 2	1	1.92 (1.13–3.27)	2.29 (1.36–3.86)	3.39 (2.08–5.54)	<0.001
Adjusted OR (95% CI), Model 3	1	1.95 (1.14–3.34)	2.36 (1.40–3.98)	3.29 (2.00–5.43)	<0.001
Adjusted OR (95% CI), Model 4	1	1.95 (1.13–3.35)	2.32 (1.37–3.93)	3.12 (1.89–5.16)	<0.001

Results from multivariate Logistic regression analysis are presented using the combined data from the two-phase independent study.

Model 1, adjusted for age, sex, and BMI;

Model 2, adjusted for Model 1, smoking, alcohol drinking, hypertension and family history of diabetes;

Model 3, adjusted for Model 2, TC, TG and HDL-C;

Model 4, adjusted for Model 3, CRP.

### ROC curves and corresponding AUCs for IGR using models without or with plasma HO-1 levels

**Figure 2:**
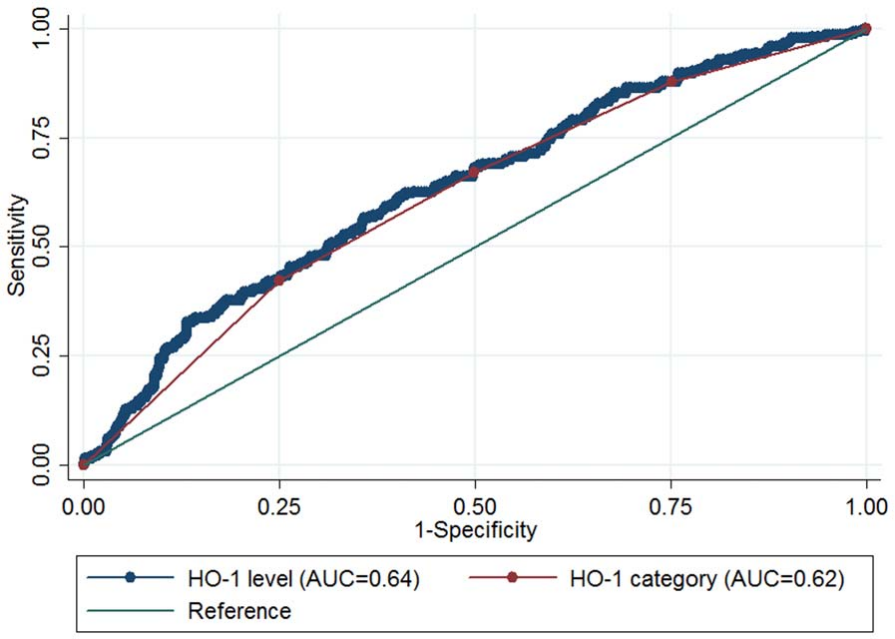
ROC curves and corresponding AUCs for IGR using plasma HO-1 levels or HO-1 categories. The AUCs for plasma HO-1 levels and HO-1 categories were 0.64 (0.60–0.68) and 0.62 (0.58–0.66), respectively; *P* = 0.0006 for the difference of the AUCs. Plasma HO-1 concentrations were categorized in quartiles according to the healthy control group as follows: category 1, <0.56 ng/ml; category 2, 0.56–0.98 ng/ml, category 3, 0.98–1.55 ng/ml and category 4, ≥1.55 ng/ml.

In the ROC curves from the logistic regression models, we first compared the AUCs for IGR using plasma HO-1 levels (continuous variable) with HO-1 categories (categorical variable), and we found that the model using plasma HO-1 levels yielded higher AUC for IGR than plasma HO-1 categories (AUC 0.64 (0.60–0.68) and 0.62 (0.58–0.66), respectively; *P* = 0.0006 for the difference of the AUCs) (Figure 2). Therefore, in the next step comparison between models without and with plasma HO-1, we chose plasma HO-1 levels in the form of continuous variable.

**Figure 3:**
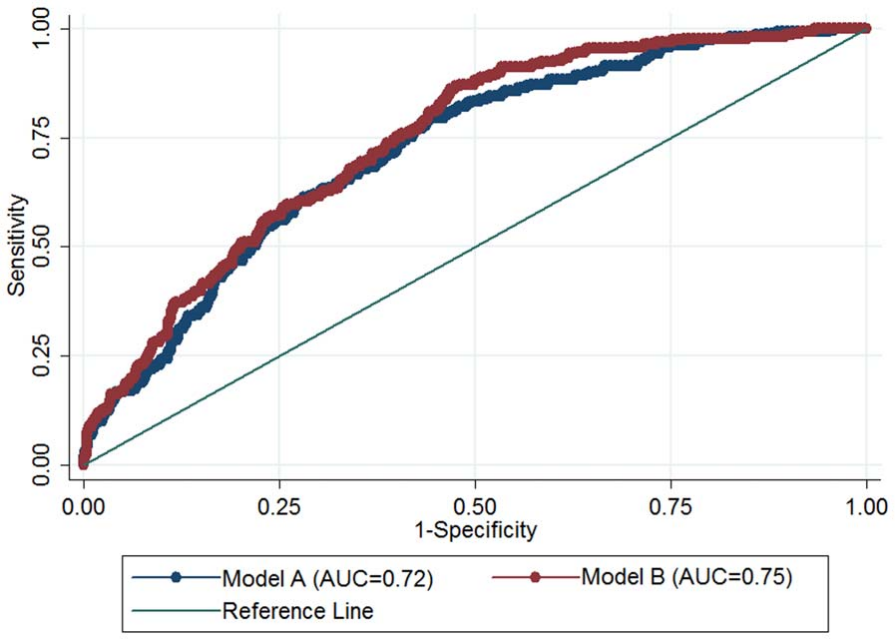
ROC curves and corresponding AUCs for IGR using models without or with plasma HO-1 levels. The AUC in a Model A, comprising age, sex, BMI, alcohol consumption, smoking, hypertention and family history of diabetes, was 0.72 (95% CI, 0.69–0.76) and that in Model B (Model A plus plasma HO-1 levels) was 0.75 (95% CI, 0.71–0.78); *P* = 0.026 for the difference of the AUCs.

As shown in Figure 3, the AUC for a model with known risk factors (Model A), comprising age, sex, BMI, alcohol consumption, smoking, hypertention and family history of diabetes, was 0.72 (95% CI 0.69–0.76) for IGR. However, when plasma HO-1 concentration was added to the model (Model B, including Model A plus HO-1), the AUC was significantly increased to 0.75 (95% CI 0.71–0.78; *P* = 0.026 for the difference of the AUCs).

## Discussion

The current study indicates that plasma HO-1 concentration is elevated in individuals with IGR in comparison with healthy controls, and it is significantly associated with odds of IGR. The association between plasma HO-1 concentrations and IGR retains rather consistent under adjustment for possible confounding factors including age, sex, BMI, smoking, alcohol drinking, hypertension, family history of diabetes, lipid profiles and CRP, which makes plasma HO-1 more convincing as an emerging independent marker for IGR.

IGR, also referred to pre-diabetes, has been recognized as a metabolic intermediate state between a “normal” state and T2DM [15], [17]. It should be noted that the IGR state has both progressive and reversible properties. IGR people shows approximately 10-fold higher risk of T2DM [1], and on the other hand, the onset of T2DM in individuals with IGR can be delayed or prevented through lifestyle modifications [18], [19]. Therefore, it is crucial to elucidate the underlying mechanisms of IGR and to develop biomarkers to understand the role of corresponding mechanisms in human.

Under high glucose exposure, HO-1 gene expression and enzyme activities in the islets are elevated remarkably in parallel with hyperglycemia-induced intracellular peroxide levels [20], [21], prior to the elevation of classical antioxidant enzymes (e.g., superoxide dismutase, catalase and glutathione peroxidase) [22]. In addition, HO-1 mediates the anti-inflammatory effect of interleukin-10 through a p38 mitogen-activated protein kinase-dependent pathway [23].

Recently, it has been found that HO-1 is present and detectable in serum or plasma samples and serves as a systemic stress marker [12], [24]. Elevated circulating HO-1 levels are subsequently found in several oxidative stress-related illness conditions, such as in chronic silicosis [12], T2DM [9], acute myocardial infarction [11], coronary microvascular dysfunction [10], Parkinson's disease [13], and critically ill patients [14]. Similar to our previous report [9], we found that plasma HO-1 concentrations were significantly correlated with fasting plasma glucose, 2 hour OGTT glucose, HOMA-beta and HOMA-IR in this population. Taken together with our previous study [9], the study shows an association that could suggest that HO-1 is responsive to high blood glucose, even if under moderate hyperglycemia in the form of IGR. The current study also suggests that plasma HO-1 concentration can significantly improve the discriminative value for IGR. If the utility of HO-1 be confirmed in prospective cohort studies, it might be incorporated into established panel of biomarkers [25] to help further improve the predictive value for IGR and T2DM.

There are several limitations to this study. First, the current case-control study design could not allow examining the causal relationship between plasma HO-1 and IGR, which remains to be confirmed in further prospective cohort studies. We also cannot directly evaluate the predictive value for IGR, thus we only evaluate the discriminative value instead. Second, we did not have information on diet and physical activity among the participants, while it is possible that individuals with IGR have a different dietary and exercise habits compared to controls. Whether such difference confounds our results remains unknown. Third, since it has been demonstrated that changes in 2-h postload glucose develops years prior to worsening in fasting glucose [26], IFG and IGT might represent not only different types of glucose abnormality, but also different stages along the same line of abnormality. However, we did not perform a separate analysis for IFG group and IGT group, because there were only 21 IFG subjects (8%) in our IGR cases group. Whether there is a significant difference in association between HO-1-IFG and HO-1-IGT merits further investigation. Fourth, diabetic comorbidities may occur in a small portion of patients with prediabetes. Although we excluded those with known cardiovascular disease, it was difficult to eliminate potential bias because elevated circulating HO-1 levels may be a result of diabetic comorbidities. Fifth, all participants in this study were of Chinese Han ethnicity, which minimizes the confounding effects by ethnic background. Whether these results can be generalized to other populations need to be studied further.

In conclusion, elevated plasma HO-1 concentration is significantly associated with increased ORs for IGR. However, its clinical utility should be validated in further studies, especially in prospective cohort studies.
